# Modified Z-plasty of the Patellar Ligament with Reinforcement of the Quadriceps Tendon in the Treatment of Patella Baja

**DOI:** 10.1055/s-0043-1770967

**Published:** 2024-04-19

**Authors:** Tiago António Almeida Orange Costa, Francisco Bernardes, José Miradouro, Joana Pereira, Pedro Barreira, João Carvalho

**Affiliations:** 1 Centro Hospitalar do Tâmega e Sousa, Penafiel, Portugal

**Keywords:** arthroplasty, replacement, knee, knee joint, patella, patellar ligament, tendons

## Abstract

Patella baja is an infrequent knee pathology, but it is limiting due to joint stiffness and localized pain in the anterior region of the knee. It may occur after trauma, prolonged immobilization or local surgical intervention. The striking pathological finding is the shortening and increase in thickness of the patellar ligament. Several surgical techniques have been described for its treatment, and there is no standardized treatment. We describe the case of a 73-year-old female patient who presented with knee stiffness, significant functional deficit, and patella baja after total knee arthroplasty. She underwent a recently described soft tissue surgical procedure, with excellent functional evolution, improving from a Lysholm Knee Score of 16 to 81 points, allowing early mobilization and return to daily life activities.

## Introduction

Patella baja is a pathology defined as the decrease in the distance between the inferior apex of the patella and the proximal articular surface of the tibia, in a patient with symptoms of gonalgia and mobility deficit. 1 2 3 4 5

It is associated with chronic gonalgia, mechanical conflict of the patella, quadriceps muscle insufficiency and decreased range of motion. 1 2 3 4 5 6 7 8 9

Key anatomopathological findings are shortening and increase in ligament thickness, intra-articular fibrosis and Hoffa's fat, tissue retraction and weakness or dysfunction of the quadriceps muscle. 1 2 3 4 5 6 7 8 9

There are several radiological methods to assess patellar height: Insall-Salvati ratio, Blackburne-Peel index and Caton-Deschamps index. 1 4 5 In describing this case, the authors used the Caton-Deschamps index, with values < 0.6 indicating the existence of patella baja. 1 4 5 7

Both surgical procedures and trauma and knee immobilization predispose to the development of patella baja. 1 2 3 4 5 6 7 8 Recent studies show incidence rates of patella baja after total knee arthroplasty of up to 38%. 1 4 7 8 9 This high incidence rate is due to excessive excision of Hoffa's fat, aggressive manipulation of the patellar ligament and release of the lateral patellar retinaculum, which in turn lead to ischemic phenomena, anomalous scarring and tendinous retraction. 1 2 3 7 8 9

There are multiple surgical procedures described for the treatment of this pathology with an impact on blood supply, on the vector of forces and on patellar tilt, with the aim of correcting the underlying etiology and recovering the normal height of the patella, but none is the standard treatment. 1 2 3 6 8 9 The objective of this clinical case was to describe a surgical technique used to correct the shortening and thickness of the patellar ligament.

## Case Report

**Fig. 1:**
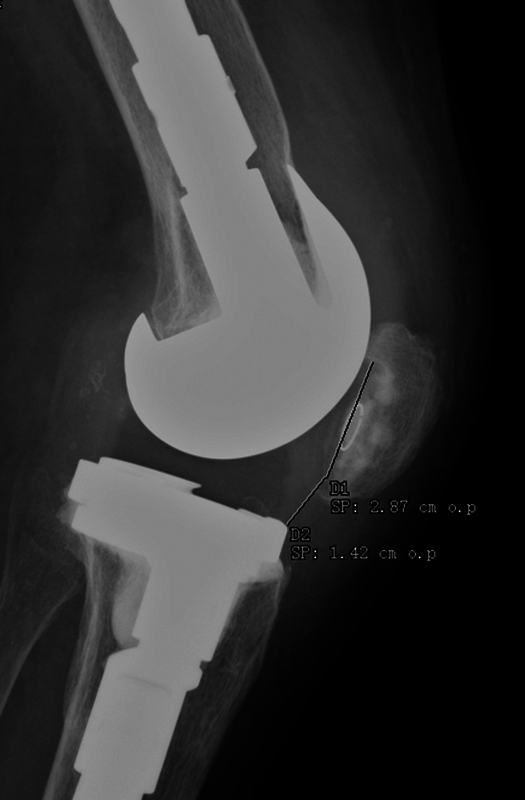
Lateral radiograph of the knee showing the preoperative Caton-Deschamps index 0.49.

A 73-year-old patient presented with knee stiffness with flexion deficit and, radiologically, patella baja after total arthroplasty of the right knee. The functional limitation, according to the Lysholm Knee Score, was 16 points, with a maximum flexion of 45°, pain and weakness of the extensor apparatus, being resistant to conservative treatment. Initially, the Caton-Deschamps index was 0.49 ( Fig. 1 ).

## Surgical Technique and Rehabilitation

The patient underwent a combined surgical procedure of patellar ligament repair for lengthening and reinforcement with part of the quadriceps tendon. The polyethylene on the upper articular surface of the tibia was replaced with a thinner one, and the patellar component was removed.

**Fig. 2:**
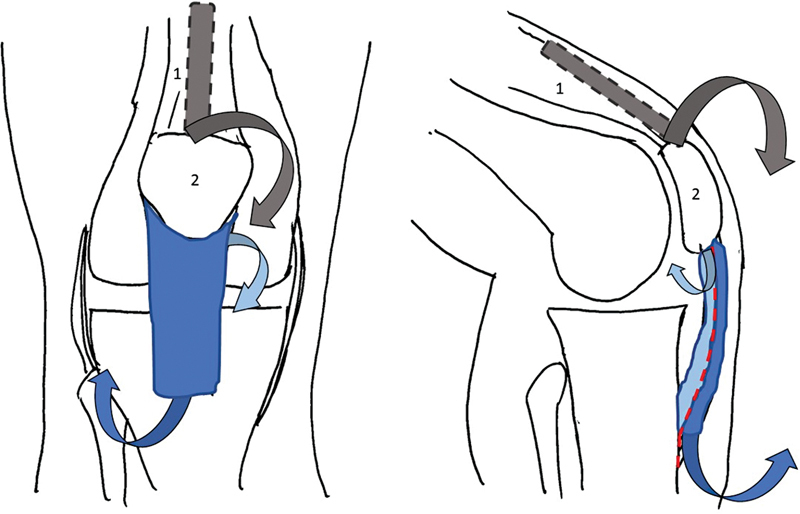
Scheme of the surgical procedure: patellar ligament was divided in two, according to its thickness. The anterior bundle (dark blue) was detached anteriorly at the level of the anterior tibial tuberosity, the posterior bundle (light blue) was detached at the level of the distal apex of the patella. Also note the acquisition of a bundle of the quadriceps tendon (gray). (1–Quadriceps Tendon; 2–Patella; 3–Patellar Ligament).

**Fig. 3:**
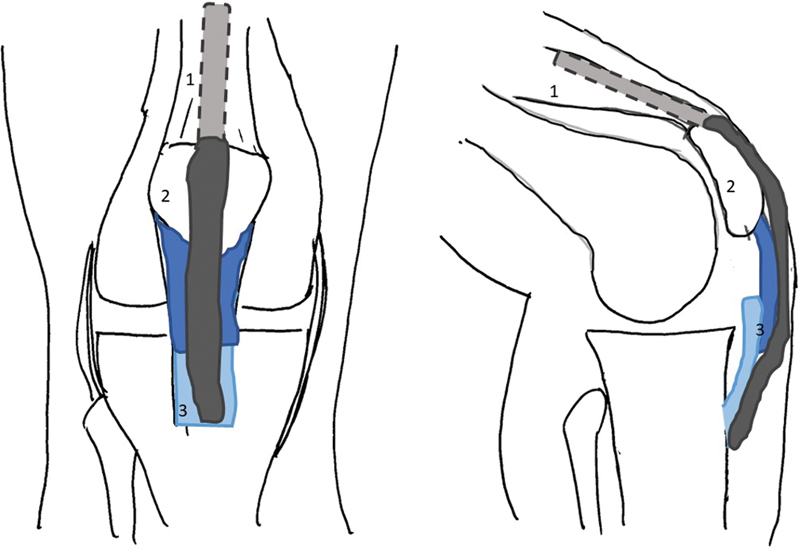
Scheme of the surgical procedure: suture of the two bundles of the patellar ligament (dark blue and light blue), with at least 5 mm of overlap. Note the ligament reinforcement with a quadriceps tendon bundle (dark grey). (1–Quadriceps Tendon; 2–Patella; 3–Patellar Ligament)

**Fig. 4:**
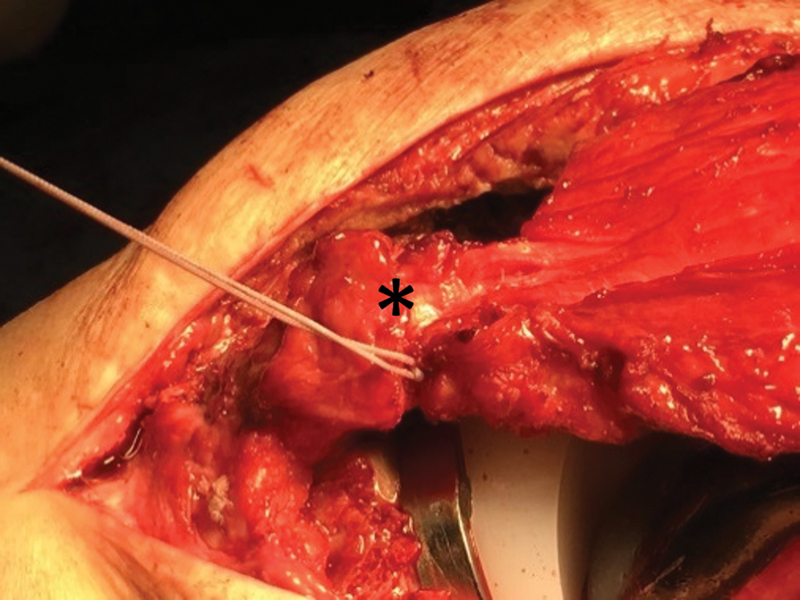
Intraoperative image demonstrating the suture of the two bundles of the patellar ligament, with at least 5mm of overlap.

**Fig. 5:**
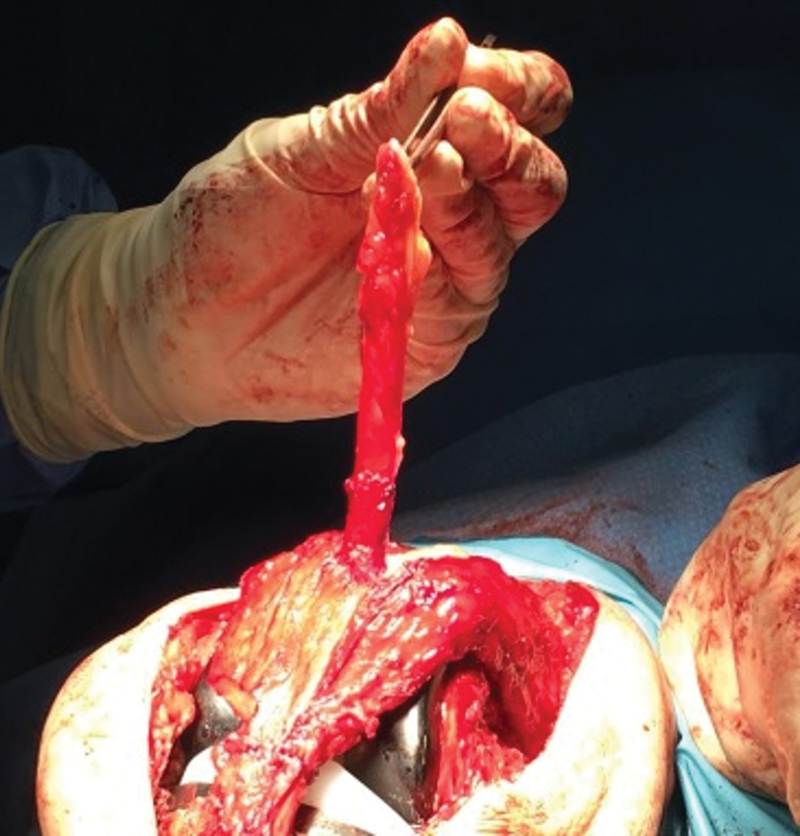
Intraoperative image demonstrating mobilization of the quadriceps tendon bundle.

**Fig. 6:**
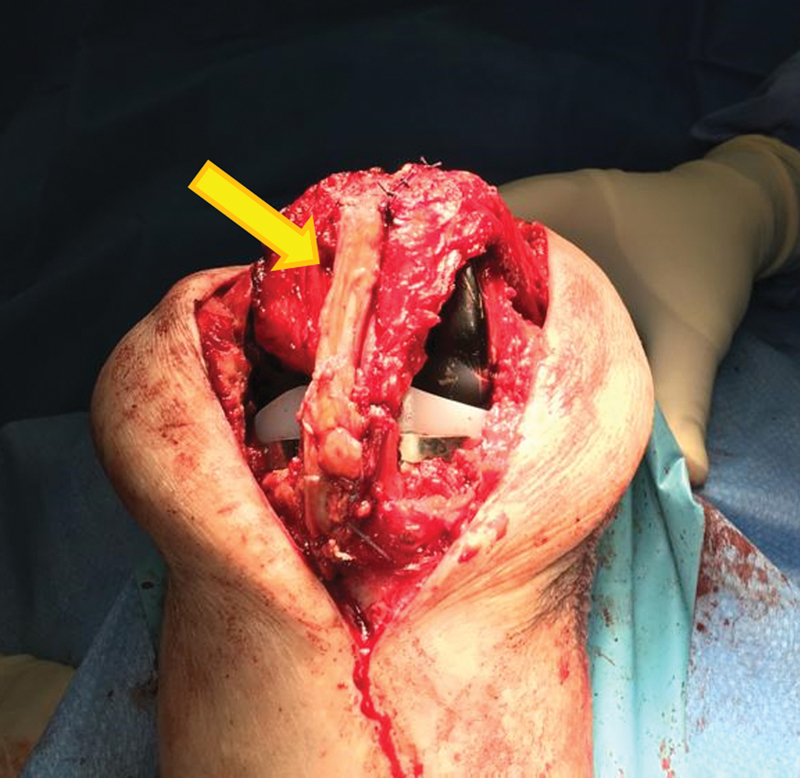
Intraoperative image demonstrating reinforcement of the patellar ligament with a quadriceps tendon bundle. (yellow arrow)

As the patellar ligament thickened, the technique involved dividing the ligament in its thickness, obtaining an anterior and a posterior component of equal thickness. 6 The anterior component was detached, distally, at the level of the anterior tibial tuberosity, and the posterior component, proximally, at the level of the patella 6 ( Fig. 2 ). With the knee at 90° of flexion, the two ligament ends were fixed with at least 5mm of overlap with absorbable suture 6 ( Figs. 3 and 4 ). A bundle of the superficial quadriceps tendon, approximately 1cm wide and 10cm long, was isolated ( Fig. 5 ) and mobilized distally to reinforce the patellar ligament 6 ( Fig. 6 ). Quadriceps excision site was reinforced with absorbable sutures, presenting low morbidity. 6

**Fig. 7:**
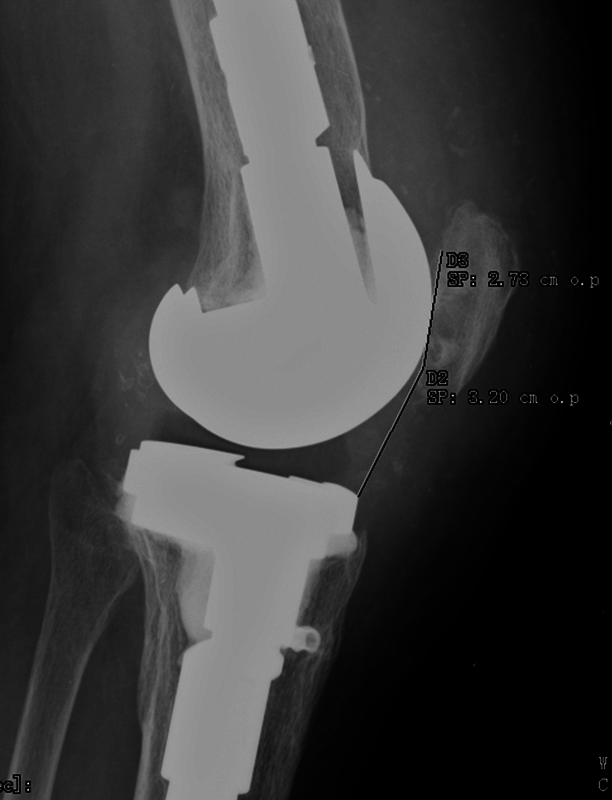
Lateral radiograph of the knee showing a Caton-Deschamps index of 1.17 postoperatively.

During the first month, immediate mobilization and rehabilitation treatment were necessary to strengthen the quadriceps and prevent relapse of the patella baja, being limited to partial load and maximum flexion of 45°. 6 9 Muscle strengthening and joint rehabilitation was maintained with the support of physical medicine and rehabilitation for three months. At six months of follow-up, the functional results were excellent, with functional results of Lysholm Knee Score of 81 points, maximum flexion of 110° and Caton-Deschamps index of 1.17 ( Fig. 7 ). The patient resumed her daily activities after the first month after the surgery.

## Discussion

Patella baja is a devastating complication of knee trauma or surgery, and early diagnosis and treatment are essential for the best functional outcome. 1 2 3 4 5 6 7 8

There are several surgical techniques described for correction of soft and bony parts, however there is no standard treatment for this pathology. 1 2 3 6 8 9

Proximal mobilization of the anterior tibial tuberosity restores patellar height, but is not recommended when there is quadriceps muscle dysfunction and does not correct the shortening and thickness of the patellar ligament. 1 2 3 4 5 6 9 10

There are, however, several options for stretching the patellar ligament, such as the use of autologous grafts or allografts from the hamstring tendons or bone-tendon-bone and stretching of the patellar ligament using the Ilizarov technique. 1 2 3 4 5 6 7 8 9 10 More recently, modified Z-plasty has been described, which has some advantages compared to other techniques. 3 4 5 6 7 9

Modified Z-plasty has the advantages of obtaining greater elongation and maintenance of alignment of tendon fibers, keeping the vector of forces and blood vascularization unaltered. 6 Other advantages of this surgical technique are the prevention of subcutaneous and tendon defects, which leads to a lower risk of complications and early rehabilitation. 6

According to the existing literature, the clinical result of the presented patient is similar to other Z-plasty variants, with the advantage of maintaining the alignment of tendinous fibers and greater overlap between the two ligament tops, not altering the vector of forces and reducing the risk bankruptcy. 1 2 3 4 5 6 7 8 9 10 Compared to bone procedures, the surgical approach is less aggressive, rehabilitation and mobilization are earlier, and it also presents a lower risk of failure. 1 2 3 4 5 6 7 8 9 10

There are several surgical techniques for the treatment of patella baja, however there is no standard treatment. The surgical technique presented has the advantages of approaching the underlying pathology, ease of execution and reproducibility, mobilization, recovery and return to daily activities after the first month after surgery, as described in the case presented.
