# Supplementary material for: Modified Z-plasty of the Patellar Ligament with Reinforcement of the Quadriceps Tendon in the Treatment of Patella Baja
Source: Rev Bras Ortop (Sao Paulo). 2024 Apr 19;59(Suppl 1):e123–6. doi: 10.1055/s-0043-1770967 (PMC11254429; doi:10.1055/s-0043-1770967)
Supplement: Related texts

# Research article: Plastia em Z modificada do ligamento patelar com reforço de tendão quadricipital no tratamento da patela baixa

**Keywords:** articulação do joelho, artroplastia do joelho, ligamento patelar, patela, tendões

## Abstract

A patela baixa é uma patologia do joelho pouco frequente, mas limitante devido à rigidez articular e dor localizada na região anterior do joelho. Pode decorrer após trauma, imobilização prolongada ou intervenção cirúrgica local. O achado patológico marcante é o encurtamento e o aumento na espessura do ligamento patelar. Foram descritas várias técnicas cirúrgicas para o seu tratamento, não existindo nenhum tratamento padronizado. Descrevemos o caso de uma doente de 73 anos de idade que apresentava rigidez do joelho, deficit funcional importante e patela baixa após artroplastia total do joelho. Foi submetida a um procedimento cirúrgico de partes moles recentemente descrito, com excelente evolução funcional, melhorando de um *Lysholm Knee Score* de 16 para 81 pontos, permitindo a mobilização e regresso às atividades de vida diárias precocemente.

## Introdução

A patela baixa é uma patologia definida como a diminuição da distância entre o ápice inferior da patela e a superfície articular proximal da tíbia, num doente com sintomas de gonalgia e deficit de mobilidade. 1 2 3 4 5

Está associada a gonalgia crónica, conflito mecânico da patela, insuficiência muscular quadricipital e diminuição da amplitude articular. 1 2 3 4 5 6 7 8 9

Os achados anatomopatológicos chave são o encurtamento e aumento da espessura ligamentar, fibrose intraarticular e da gordura de Hoffa, retração tecidular e fraqueza ou disfunção do músculo quadricipital. 1 2 3 4 5 6 7 8 9

Existem vários métodos radiológicos para avaliar a altura patelar: índice *Insall-Salvati* , índice *Blackburne-Peel* e o índice *Caton-Deschamps* . 1 4 5 Na descrição deste caso, os autores utilizaram o índice *Caton-Deschamps* , sendo que valores <0,6 indicam a existência de patela baixa. 1 4 5 7

Tanto os procedimentos cirúrgicos, como o trauma e imobilização do joelho predispõem ao desenvolvimento de patela baixa. 1 2 3 4 5 6 7 8 Estudos recentes mostram taxas de incidência de patela baixa após artroplastia total do joelho de até 38%. 1 4 7 8 9 Esta elevada taxa de incidência é devido à excisão excessiva da gordura de Hoffa, manipulação agressiva do ligamento patelar e libertação do retináculo lateral da patela, que por sua vez conduzem a fenômenos isquêmicos, cicatrização anômala e retração tendinosa. 1 2 3 7 8 9

Existem múltiplos procedimentos cirúrgicos descritos para o tratamento desta patologia com impacto no aporte sanguíneo, no vetor de forças e no *tilt* patelar, com o objetivo de corrigir a etiologia de base e recuperar a altura normal da patela, mas nenhum é o tratamento padrão. 1 2 3 6 8 9 O objetivo deste caso clínico foi descrever uma técnica cirúrgica utilizada para a correção do encurtamento e espessura do ligamento patelar.

## Relato de Caso

Paciente de 73 anos de idade que apresentava rigidez do joelho com deficit de flexão e, radiologicamente, patela baixa após artroplastia total do joelho direito. A limitação funcional, segundo o *Lysholm Knee Score* , era de 16 pontos, com máximo de flexão de 45°, dor e fraqueza do aparelho extensor, sendo resistentes ao tratamento conservador. Inicialmente, o índice de *Caton-Deschamps* era de 0,49 ( Fig. 1 ).

**Fig. 1:** Radiografia lateral do joelho a evidenciar o índice *Caton-Deschamps* 0,49 no pré-operatório.

## Técnica Cirúrgica e Reabilitação

A doente foi submetida a um procedimento cirúrgico combinado de plastia do ligamento patelar para alongamento e reforço com parte do tendão quadricipital. Ainda se procedeu à substituição do polietileno da face articular superior da tíbia, por um de menor espessura e o componente patelar foi removido.

Como o ligamento patelar se encontrava espessado, a técnica envolveu a divisão do ligamento na sua espessura, obtendo-se um componente anterior e outro posterior de igual espessura. 6 O componente anterior foi desinserido, distalmente, ao nível da tuberosidade anterior da tíbia e o componente posterior, proximalmente, ao nível da patela 6 ( Fig. 2 ). Com o joelho a 90° de flexão, fixaram-se os dois topos ligamentares com, pelo menos, 5 mm de sobreposição com sutura absorvível 6 ( Figs. 3 e 4 ). Um feixe do tendão quadricipital superficial, com cerca de 1 cm de largura e 10 cm de comprimento, foi isolado ( Fig. 5 ) e mobilizado distalmente para reforço do ligamento patelar 6 ( Fig. 6 ). O local de excisão quadricipital é reforçado com suturas absorvíveis, apresentando baixa morbilidade. 6

**Fig. 2:** Esquema do procedimento cirúrgico: ligamento patelar foi dividido em dois, segundo a sua espessura. O feixe anterior (azul-escuro) foi desinserido anteriormente ao nível da tuberosidade anterior da tíbia, o feixe posterior (azul-claro) foi desinserido ao nível do ápice distal da patela. Nota-se ainda a obtenção de feixe do tendão quadricipital (cinzento). (1–Tendão Quadricipital; 2–Patela; 3–Ligamento Patelar).

**Fig. 3:** Esquema do procedimento cirúrgico: sutura dos dois feixes do ligamento patelar (azul-escuro e azul-claro), com pelo menos 5 mm de sobreposição. De notar o reforço ligamentar com feixe do tendão quadricipital (cinzento-escuro). (1–Tendão Quadricipital; 2–Patela; 3–Ligamento Patelar)

**Fig. 4:** Imagem intraoperatória a demonstrar a sutura dos dois feixes do ligamento patelar, com pelo menos 5 mm de sobreposição.

**Fig. 5:** Imagem intraoperatória a demonstrar a mobilização do feixe do tendão quadricipital.

**Fig. 6:** Imagem intraoperatória a demonstrar o reforço do ligamento patelar com feixe do tendão quadricipital. (seta amarela)

Durante o primeiro mês, a mobilização imediata e o tratamento de reabilitação foram necessários para o fortalecimento quadricipital e evitar a recidiva de patela baixa, estando limitado a carga parcial e flexão máxima de 45°. 6 9 O fortalecimento muscular e reabilitação articular foi mantido com o apoio da medicina física e de reabilitação durante três meses. Aos seis meses de seguimento, os resultados funcionais foram excelentes, com resultados funcionais de *Lysholm Knee Score* de 81 pontos, flexão máxima de 110° e índice *Caton-Deschamps* de 1,17 ( Fig. 7 ). A doente retomou as suas atividades de vida diária após o primeiro mês pós-cirúrgico.

**Fig. 7:** Radiografia lateral do joelho a evidenciar o índice *Caton-Deschamps* de 1,17 no pós-operatório.

## Discussão

A patela baixa é uma complicação devastadora do trauma ou cirurgia do joelho, sendo essencial o diagnóstico e tratamento precoces, para o melhor resultado funcional. 1 2 3 4 5 6 7 8

Existem várias técnicas cirúrgicas descritas para correção de partes moles e ósseas, contudo não existe um tratamento padrão para esta patologia. 1 2 3 6 8 9

A mobilização proximal da tuberosidade anterior da tíbia restaura a altura da patela, mas não é aconselhada quando existe disfunção do músculo quadricipital e não corrige o encurtamento e a espessura do ligamento patelar. 1 2 3 4 5 6 9 10

Há, no entanto, várias opções para o alongamento do ligamento patelar como o uso de enxertos autólogos ou aloenxertos dos tendões isquiotibiais ou osso-tendão-osso e o alongamento do ligamento patelar utilizando a técnica de Ilizarov. 1 2 3 4 5 6 7 8 9 10 Mais recentemente foi descrita a plastia em Z modificada que apresenta algumas vantagens em comparação com as outras técnicas. 3 4 5 6 7 9

A plastia em Z modificada tem as vantagens de obter maior alongamento e a manutenção do alinhamento das fibras tendinosas, mantendo inalterado o vetor de forças e a vascularização sanguínea. 6 Outras vantagens desta técnica cirúrgica são a prevenção de defeitos subcutâneos e tendinosos, o que leva a menor risco de complicações e a uma reabilitação precoce. 6

Segundo a literatura existente, o resultado clínico do paciente apresentado é semelhante a outras variantes de plastia em Z, com a vantagem de manter o alinhamento das fibras tendinosas e maior sobreposição entre os dois topos ligamentares, não alterando o vetor de forças e diminuindo o risco de falência. 1 2 3 4 5 6 7 8 9 10 Em comparação com os procedimentos ósseos, a abordagem cirúrgica é menos agressiva, a reabilitação e a mobilização são mais precoces, apresentando também menor risco de falência. 1 2 3 4 5 6 7 8 9 10

Existem várias técnicas cirúrgicas para o tratamento da patela baixa, contudo não existe um tratamento padrão. A técnica cirúrgica apresentada tem como vantagens a abordagem da patologia subjacente, facilidade de execução e reprodutibilidade, mobilização, recuperação e regresso às atividades de vida diárias após o primeiro mês pós-cirúrgico, como descrito no caso apresentado.
